# Isolating together during COVID-19: Results from the Telehealth Intervention Program for older adults

**DOI:** 10.3389/fmed.2022.948506

**Published:** 2022-10-11

**Authors:** Harmehr Sekhon, Paola Lavin, Blanca Vacaflor, Christina Rigas, Karin Cinalioglu, Chien-Lin Su, Katie Bodenstein, Elena Dikaios, Allana Goodman, Florence Coulombe Raymond, Marim Ibrahim, Magnus Bein, Johanna Gruber, Jade Se, Neeti Sasi, Chesley Walsh, Rim Nazar, Cezara Hanganu, Sonia Berkani, Isabelle Royal, Alessandra Schiavetto, Karl Looper, Cyrille Launay, Emily G. McDonald, Dallas Seitz, Sanjeev Kumar, Olivier Beauchet, Bassam Khoury, Stephane Bouchard, Bruno Battistini, Pascal Fallavollita, Marc Miresco, Marie-Andrée Bruneau, Ipsit Vahia, Syeda Bukhari, Soham Rej

**Affiliations:** ^1^Department of Psychiatry, McGill University, Montreal, QC, Canada; ^2^Jewish General Hospital/Lady Davis Institute, Montreal, QC, Canada; ^3^GeriPARTy Research Lab, Montreal, QC, Canada; ^4^McLean Hospital, Harvard Medical School, Boston, MA, United States; ^5^Pierre-Boucher Hospital, Longueuil, QC, Canada; ^6^Department of Medicine, McGill University, Montreal, QC, Canada; ^7^Department of Psychiatry, University of Calgary, Calgary, AB, Canada; ^8^Geriatric Clinical Research, The Centre for Addiction and Mental Health, Toronto, ON, Canada; ^9^Department of Educational and Counselling Psychology, McGill University, Montreal, QC, Canada; ^10^Department of Psychoeducation and Psychology, Université du Quebec en Outaouais, Gatineau, QC, Canada; ^11^Quebec Heart and Lung Institute, Department of Medicine, Laval University, Quebec City, QC, Canada; ^12^Interdisciplinary School of Health Sciences, University of Ottawa, Ottawa, ON, Canada; ^13^Department of Psychiatry, University of Montreal, Montreal, QC, Canada

**Keywords:** telehealth, older adults, COVID-19, isolation, stress, mental health support, depression, anxiety

## Abstract

**Background:**

A pressing challenge during the COVID-19 pandemic and beyond is to provide accessible and scalable mental health support to isolated older adults in the community. The Telehealth Intervention Program for Older Adults (TIP-OA) is a large-scale, volunteer-based, friendly telephone support program designed to address this unmet need.

**Methods:**

A prospective cohort study of 112 TIP-OA participants aged ≥60 years old was conducted in Quebec, Canada (October 2020–June 2021). The intervention consisted of weekly friendly phone calls from trained volunteers. The primary outcome measures included changes in scores of stress, depression, anxiety, and fear surrounding COVID-19, assessed at baseline, 4 and 8-weeks. Additional subgroup analyses were performed with participants with higher baseline scores.

**Results:**

The subgroup of participants with higher baseline depression scores (PHQ9 ≥10) had significant improvements in depression scores over the 8-week period measured [mean change score = −2.27 (±4.76), 95%CI (−3.719, −0.827), *p* = 0.003]. Similarly, participants with higher baseline anxiety scores (GAD7 ≥10) had an improvement over the same period, which, approached significance (*p* = 0.06). Moreover, despite peaks in the pandemic and related stressors, our study found no significant (*p* ≥ 0.09) increase in stress, depression, anxiety or fear of COVID-19 scores.

**Discussion:**

This scalable, volunteer-based, friendly telephone intervention program was associated with decreased scores of depression and anxiety in older adults who reported higher scores at baseline (PHQ 9 ≥10 and GAD7 ≥10).

## Introduction

Older adults are at higher risk of social isolation compared to younger adults (1). The COVID-19 pandemic has further amplified this problem with lockdowns and social distancing measures designed to decrease risk of infection in older adults. As a result, an unprecedented number of older adults have found themselves isolated and disconnected from others. Recent literature shows that social disconnection in older adults is associated with higher levels of stress, suicide ideation, and self-harm (2). As well, social isolation in older adults is associated with an increased risk of medical and psychiatric comorbidity (3, 4), premature mortality (5) and poor quality of life (6), relative to older adults who are not isolated. The scarcity of resources, the need for scalable, low-cost and effective interventions to help care for this population has never been greater. To address this need, there has been a large uptake in the use of technology to facilitate access to services (7). Telehealth has demonstrated evidence as a modality for reducing depression (8) and anxiety (9) in older adults. Technology has also been found to be successful in connecting socially isolated older adults (10–12) as the vast majority have access to a phone (e.g., mobile phone, home phone) (13, 14). However, due to the digital divide, many older adults may have difficulty using more modern devices, limiting their capacity to engage in virtual meetings and therefore putting them at risk of heightened social isolation relative to the general population.

Another challenge in the development and provision of services to isolated older adults is the limited availability of health resources and trained personnel. Fortunately, lay-volunteer interventions have been shown to improve depression and other mental health symptoms in older adults, making this a scalable approach (15). As such, telephone-based support with volunteers can be a potentially rapid, inexpensive, and convenient intervention option for the urgently required support for isolated older adults. This led to the development of the novel and scalable Telehealth Intervention Program for older adults (TIP-OA) in March 2020 by the GeriPARTy research group (16). TIP-OA is a friendly phone call service (providing social interaction for isolated older adults) that now serves >800 older adults with 350 trained volunteers in the province of Quebec, Canada. The aim of this multilingual (>15 languages, e.g., Hebrew, Chinese, Spanish, Arabic, Urdu, Punjabi, Italian, Russian, Greek, etc.) program is for volunteers to provide social interactions, connect older adults with existing community resources/networks, and help them navigate and access online resources (e.g., grocery delivery, pharmacy refills and delivery). Although comparable telehealth programs for older adults have been set up in North America and Europe throughout the pandemic, few have assessed their effectiveness on participants' mental health by means of empirical research. Of the few that did, most focused on cognitive functioning (17). The primary objective of this study was to evaluate the effectiveness of TIP-OA in reducing participants' stress from baseline to 8-week follow-up. The secondary objective was to evaluate effectiveness in improving depression, anxiety, and fear associated with COVID-19 scores. We hypothesised that participants would report decreased scores of stress, anxiety, depression, and fear of COVID-19 at 8-week follow-up compared to baseline.

## Methods

### Study design

This was a prospective, longitudinal 8-week study of a large-scale volunteer-based telehealth intervention program for older adults (TIP-OA). Data collection occurred at baseline (prior to, or after receiving only one phone call from a volunteer), at 4-weeks (±1 week), and at the 8-week mark (±1 week), which was the primary study endpoint.

### Ethics

The protocol was conducted in accordance with the Declaration of Helsinki and approved by the Jewish General Hospital Research Ethics Committee on September 24, 2020. This study has been registered on clinicaltrials.gov, registration #NCT04523610.

### Sample size and recruitment

Over 270 consecutive prospective TIP-OA program users were contacted contacted and 111 consented to participate in this study. Recruitment took place October 2020–February 2021 and participants were followed until 8-week follow-up. Older adults were referred to the TIP-OA program either through community partners (*n* = 11) or by providing consent to other referral sources such as their community workers or clinicians in long-term care facilities, geriatric psychiatry, geriatric medicine, family medicine clinics, local public primary care centres, or as self-referrals through a 1–800 number. The research team then reached out to them by phone to confirm eligibility and obtain verbal informed consent to participate in the study. “Risk rating” was coded as colours assigned by clinicians after phone assessment of confusion, psychotic thoughts, depression/anxiety, suicidality, functional impairment, COVID distress, and other conditions. Participants with no/mild ratings were coded as green (low risk), 2+ moderate ratings as orange (medium risk), and 1+ severe rating as red (high risk) (16). For more details, please refer to the previous publication by Dikaios et al. (16).

### Inclusion/exclusion criteria

The inclusion criteria for this study was: (1) TIP-OA users aged ≥60 living in Quebec, and (2) spoke English or French. Individuals who had psychotic symptoms, severe hearing impairment, or active suicidal ideation were excluded.

### Intervention

TIP-OA involved weekly, friendly phone calls from trained volunteers to older adults (aged ≥60), including those experiencing mental health/cognitive issues. The volunteer-based telehealth calls primarily provided friendly social interaction lasting between 5 and 90 min (average 30 min), depending on participants' preferences. Volunteers inquired about clients' general wellbeing, provided updated public health recommendations about COVID-19, conducted a brief needs assessment (e.g., food delivery, medication from their pharmacy, transportation), offered support accessing community resources and fostered social connections through active listening, validation, and conversation. A total of 82 TIP-OA volunteers participated in the study. The majority (*n* = 65, 79%) were undergraduate/graduate students//employees from community organizations or retired healthcare professionals. A smaller proportion of volunteers (*n* = 17, 21%) were other members from the community. For protocol details, please refer to the publication by Dikaios et al. (16).

### Volunteer training

Telehealth Intervention Program-OA volunteers underwent a rigorous application and selection process. Selected candidates attended a 2-h TIP-OA training session conducted by clinicians through Zoom. Each training group was composed of 4–6 trainees and led by two trainers, to ensure optimal opportunity for interactive training. Moreover, a detailed training manual was provided, which covered an overview of the program, sample conversations, client confidentiality, and an extensive list of community resources (e.g., grocery and pharmacy delivery services). Twice a week drop-in follow-up sessions were offered to all volunteers with the purpose of debriefing and receiving support from the clinicians and trainers. For protocol details, please refer to the publication by Dikaios et al. (16).

## Measures

### Demographic variables

The following variables were collected: age, sex, living setting (e.g., living alone, long-term care), neighbourhood of residence, marital status, highest level of education, language(s) spoken, ethnicity, and baseline risk level (colour coding described in Dikaios et al. (16)). Additional information about digital access and literacy was obtained (e.g., access to and ability to use telephone, computer, internet, Facetime/Zoom).

### Primary outcome measure

The Perceived Stress Scale (PSS) is a 14-item scale that was used to measure stress (18), inquiring how participants felt in the past month and the degree to which life events were experienced and appraised as stressful, with responses ranging from 0 (never) to 4 (very often) (18).

### Secondary outcomes measures

The *Patient Health Questionnaire-9 (PHQ-9) is* a 9-item questionnaire that was used to measure depression symptom severity (19). The *Generalized Anxiety Disorder-7 (GAD-7)* is a 7-item scale that was used to measure anxiety symptom severity (20).

The *COVID Fear Scale is a* 18-item scale that was used to measure the participants' anxiety, fear and concern around the current pandemic. Items included: “Fear that I will be infected” and “Worry if I will be assigned to COVID wards if hospitalized” (21).

### Subgroups

The higher-stress subgroup consisted of participants with a PSS score ≥14 (indicative of moderate stress) (18). The higher-depression subgroup consisted of participants with a score of PHQ-9 ≥10 (indicative of moderate depression) (19). The higher-anxiety subgroup consisted of participants with a score of GAD-7 ≥10 (indicative of moderate anxiety) (20).

## Data analysis

Normality was tested using the Kolmogorov–Smirnov normality test (22). To evaluate the effectiveness of TIP-OA in reducing scores from baseline to 8-week follow-up, paired *t*-tests (23) were performed for all outcomes (PSS, PHQ-9, GAD-7, COVID fear scale). Two-tailed *p*-values < 0.05 were considered as statistically significant. Last observation carried forward (LOCF) using 4-week data was used to handle missing 8-week data (16). All statistical analyses were performed using SPSS (version 28.0; SPSS, Inc., Chicago, IL).

## Results

### Study sample/recruitment

**Figure 1:**
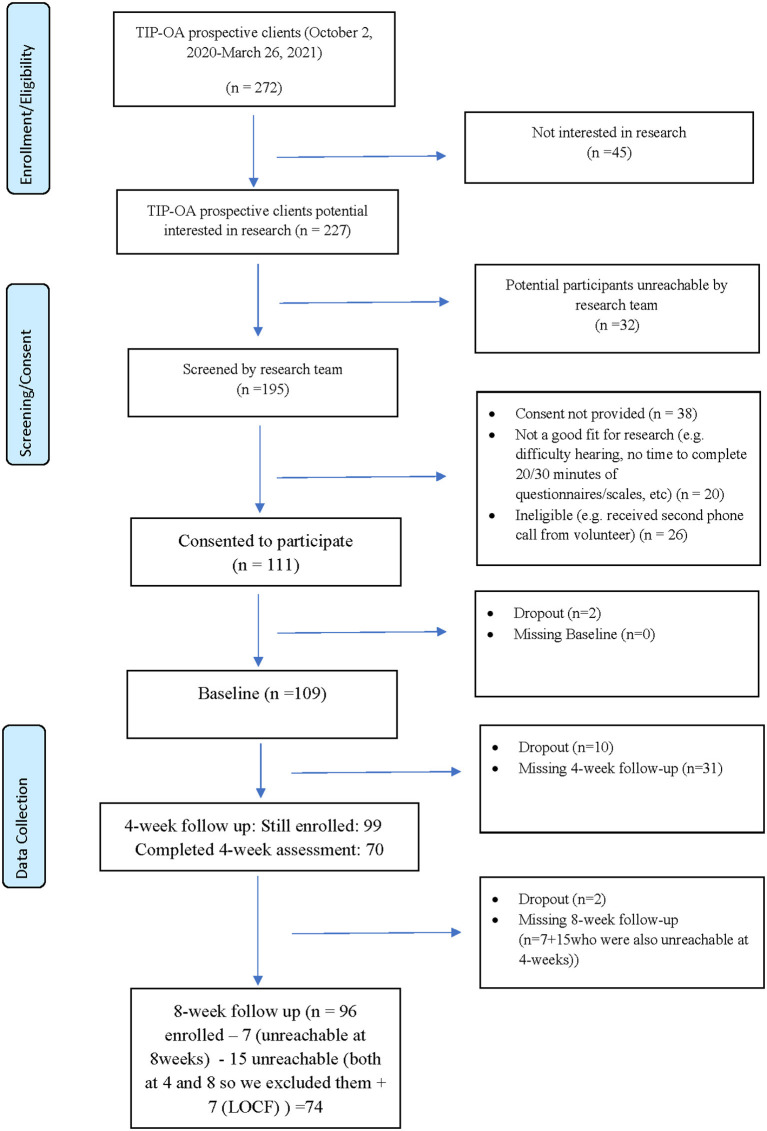
Participant flow-chart.

As described in detail in Figure 1, a total of 229 interested study candidates were contacted and 111 individuals consented (48.47%) to take part in the study. There were a total of 14 dropouts (12.61%), 78 individuals completed the intervention (6/8 weeks) and four withdrew before 6 weeks of intervention.

Participant baseline demographic characteristics are described in Table 1. Participant “Neighbourhood” was the neighbourhood of residence that participants self-reported. “Schooling” was defined by participant's highest level of education completed (elementary school graduate, high school graduate, a Bachelor's degree, or a Master's/PhD). Participant's marital status was defined by if they were currently single, married/common-law, or separated. For “languages,” regardless of participants' spoken languages, all research participants spoke either English, French, or were bilingual (spoke English and French). “Minority status” is defined as self-identifying as a visible minority. Ethnicity was defined by patients self-identifying as African, Caribbean, Caucasian, Southeast Asian, Mixed, or Other. For “living situation,” support was defined as living with another family member, a spouse, child, friend, or in shared housing. For details regarding “Risk rating,” please refer to the “Sample size and recruitment” section.

**Table 1:** Demographics and clinical characteristics.

**Baseline characteristics (*n*)**	**Mean or frequency**	**SD or %**
Age (years), mean ± SD (*n* = 82)	73.18	7.88
**Gender (*****n*** **=** **82)**		
Male	24	29.3
Female	58	70.7
**Schooling (*****n*** **=** **80)**		
Elementary school graduate	12	14.6
Highschool graduate	35	42.7
University	33	40.2
**Marital status (*****n*** **=** **82)**		
Single/separated	59	72.0
Married/common–law	23	28.0
**Languages (*****n*** **=** **82)**		
English	20	24.4
French	14	17.1
Bilingual (English and French)	48	58.5
**Minority status (*****n*** **=** **75)**		
No	63	76.8
Yes	10	12.2
Unsure	2	2.4
**Ethnicity (*****n*** **=** **74)**		
African	3	3.7
Caribbean	6	7.3
Caucasian	46	56.1
Southeast Asian	1	1.2
Mixed	4	4.9
Other	14	17.1
**Living situation (*****n*** **=** **82)**		
Alone	63	76.8
Support or living with someone	19	23.2
**Type of dwelling (*****n*** **=** **82)**		
Senior residences	12	14.7
Community dwelling	70	85.3
**Risk level (*****n*** **=** **82)**		
Red	7	8.5
Yellow	25	30.5
Green	50	61.0

Table 2 shows the results of the paired *t*-tests for all participants. The mean (SD) change score for PSS (*n* = 79) at 8 weeks was −0.58 (6.66). There were no significant differences in PSS scores between baseline and 8-weeks [18.7 ± 8.25 vs. 18.1 ± 8.76 SD, *t*_(78)_ = −0.78, *p* = 0.43, 95% CI (−2.07, 0.90), Cohen's *d* = 0.08, 95% CI for *d* (−0.13, 0.30)]. The mean (SD) PHQ-9 (*n* = 78) change score was −0.85 (4.48). There was no significant difference in PHQ-9 scores between baseline and 8-weeks [*t*_(77)_ = −1.67, *p* = 0.09, 95% CI (−1.85, 0.16), *d* = 0.18, 95% CI (−0.03, 0.41)]. The mean (SD) GAD-7 (*n* = 79) score was −0.14 (4.82). No significant differences were found in GAD-7 scores between baseline and 8-weeks [*t*_(78)_ = −0.25, *p* = 0.79, 95% CI (−1.21, 0.94), *d* = 0.02, 95% CI (−0.19, 0.24)]. The mean COVID fear (*n* = 74) change score was −0.92 (6.06). There was no significant difference in COVID Fear scores between baseline and 8-weeks [*t*_(73)_ = −1.30, *p* = 0.19, 95% CI (−2.32, 0.48), *d* = 0.15, 95% CI (−0.07, 0.38)].

**Table 2:** Mean change after 8–weeks for all participants.

**Measures**	**Mean change after 8–weeks (SD)**	***t* statistic (*df*)**	***p*–Value**	**95% confidence interval**	**Cohen's *d***	**95% confidence interval**
PSS	−0.58 (6.66)	*t*_(78)_ = −0.78	0.43	−2.07, 0.90	0.08	−0.13, 0.30
PHQ-9	−0.85 (4.48)	*t*_(77)_ = −1.67	0.09	−1.85, 0.16	0.18	−0.03, 0.41
GAD-7	−0.14 (4.82)	*t*_(78)_ = −0.26	0.79	−1.21, 0.94	0.02	−0.19, 0.24
CFS	−0.92 (6.06)	*t*_(73)_ = −1.30	0.19	−2.32, 0.48	0.15	−0.07, 0.38

PSS, Perceived Stress Scales; PHQ-9, Patient Health Questionnaire-9; GAD-7, generalized anxiety disorder-7; CFS, Covid Fear Scale.

Table 3 shows the results of the paired *t*-tests for each participant subgroup. The mean (SD) change score for PSS subgroup (*n* = 58, PSS ≥14) at 8 weeks was −1.09 (7.15). There were no significant differences in PSS subgroup scores between baseline and 8-weeks [*t*_(57)_ = −1.086, *p* = 0.25, 95% CI (−2.96, 0.79), *d* = 0.15, 95% CI (−0.10, 0.41)]. The mean (SD) change score for the PHQ-9 subgroup (*n* = 44, PHQ-9 ≥ 10) was −2.27 (4.76). There was a significant difference in PHQ-9 subgroup scores between baseline and 8-weeks [*t*_(43)_ = −3.17, *p* = 0.00, 95% CI (−3.71, −0.82), with a medium effect size of *d* = 0.47, 95% CI (0.16, 0.78)]. The mean (SD) GAD-7 subgroup (*n* = 30, GAD-7 ≥10) change score was −1.93 (5.42). There was a difference between baseline and 8-weeks close to significance [*t*_(29)_ = −1.95, *p* = 0.06, 95% CI (−3.95, 0.09), *d* = 0.35, 95% CI (−0.01, 0.72)].

**Table 3:** Mean change after 8–weeks for subgroup participants.

**Measures**	**Mean change after 8–weeks (SD)**	***t* statistic (*df*)**	***p*–Value**	**95% confidence interval**	**Cohen's *d***	**95% confidence interval**
PSS ≥ 14	−1.09 (7.15)	*t*_(57)_ = −1.15	0.25	−2.96, 0.79	0.15	−0.10, 0.41
PHQ-9 ≥ 10	−2.27 (4.76)	*t*_(43)_ = −3.17	0.00	−3.71, −0.82	0.47	0.16, 0.78
GAD-7 ≥10	−1.93 (5.42)	*t*_(29)_ = −1.95	0.06	−3.95, 0.09	0.35	−0.01, 0.72

PSS, Perceived Stress Scales; PHQ-9, Patient Health Questionnaire-9; GAD-7, generalized anxiety disorder-7; CFS, Covid Fear Scale.

## Discussion

The aim of this study was to document the impact of a weekly friendly telephone support (TIP-OA) in improving mental health outcomes and fear of COVID-19 from baseline to 8-week follow-up. Our study found that participants did not develop higher scores of stress, anxiety, depression and fear associated with COVID-19 at 8-weeks, compared to baseline, despite the intervention time frame overlapping with stressors of the high peaks of the pandemic in Quebec. However, the subgroup of individuals with higher baseline scores of depression (PHQ-9 score ≥10) showed a statistically significant reduction in scores at 8-week follow-up [mean change score = −2.27 (±4.76), 95%CI (−3.71, −0.82), *p* = 0.00]. The subgroup of individuals with higher baseline scores of stress (PSS score ≥14) and anxiety (GAD-7 score ≥10) showed a reduction in scores at 8-week follow-up, however, this was not statistically significant. These results suggest that participants' mental health symptomatology stabilized over the 8-week intervention. These are positive findings as the intervention for this study was conducted between October 5th 2020 and June 7th, 2021, coinciding with record COVID-19 infections in Quebec (24), during which media-related stressors and isolation restrictions would have presumably been at their peak. Recent literature has shown that persistent isolation and exposure to worrying COVID-19 statistics and media coverage is associated with adverse mental health outcomes (25), The finding that participants' overall mental health scores did not significantly worsen during this time period suggests that TIP-OA may have played an important role in preventing mental health deterioration in at-risk older adults.

Furthermore, fear of COVID-19 scores for all participants decreased at 8-weeks, compared to baseline. Although this was not a statistically significant finding, it aligns with recent literature showing that being able to openly discuss your fear about COVID-19 with an informed individual who is able to provide accurate information decreases fear of COVID. (26) Besides providing regular friendly phone calls that increase social connection, the TIP-OA program showed two major strengths: (1) the program was able to provide reliable information regarding COVID-19 and government regulations and (2) it was able to facilitate access to community resources (e.g., connect with community programs, food/grocery delivery services, pharmacy delivery services), ultimately helping to mitigate the negative impact of the COVID-19 pandemic. A potential benefit of this intervention was that it could also decrease the burden on the healthcare system as older adults at higher risk of social isolation are associated with a 50% higher healthcare cost and increased resource requirements (27–31).

This study found that the subgroup of individuals with high depressive scores at baseline showed the most significant improvement at 8-week follow-up. Individuals with high depressive scores are known to be most at risk for developing clinical depression which is associated with higher mortality, morbidity, and healthcare (32). Due to the preventative approach, it is possible that the program might have contributed in cutting down the medical cost. Late life depression is especially associated with higher caregiver burden and distress (33). Improving the mental health of those most at risk for clinical depression is the need of the hour, especially during the pandemic with an already overburdened healthcare system (34).

Together, these results speak to the strength of the TIP-OA intervention and its potential to stabilize and improve older adults' mental health outcomes, especially for the most vulnerable older adults, by means of a community-based program that has served over 800 older adults in Quebec, from diverse socio-economic and cultural backgrounds in ≥15 languages.

### Strengths and limitations

Although an RCT design is the gold standard trial to assess the effectiveness of the intervention, the TIP-OA team opted for an uncontrolled prospective cohort study. This was meant to be able to provide the service to as many individuals as possible in a timely manner due to COVID-19's impact on mental health and social isolation. In the context of the beginning of the pandemic, having a control group in this study would have delayed access to the service for vulnerable participants with high risk for mental health decline by 8-weeks or more. Additionally, this study design decision was supported by funding avenues dedicated to enhancing accessibility to mental health services in the community. One major strength of this study is that this is a volunteer-based mental health program for older adults that does not place additional demands on the already strained healthcare system nor further burdens healthcare workers and caregivers. In fact, the program supported several social workers and community organizations serving older adults by providing the service to their clients and taking the stress off of their shoulders to deal with the emergency situation created by the pandemic.

Another strength of the TIP-OA program is that it serves individuals in >15 languages which opens a bridge to serve immigrant adults who arrived to Canada later in life and experience language barriers to access services. Future confirmatory RCTs with a larger sample size and a longer study period could be used to further evaluate changes in stress and other outcomes, the effectiveness of TIP-OA, and the impact on older adults who do not speak English/French and may be at greater risk for social isolation. In future studies, identifying if participating individuals were also caregivers may be important to assess the increased caregiver burden during the pandemic.

## Conclusion

This study has found that a friendly phone call program (TIP-OA) can help stabilize and decrease mental health symptoms in older adults, with a most pronounced effect on depression scores. TIP-OA supports the most vulnerable community-dwelling older adults who are isolated and experiencing mental health distress through a trained lay volunteer community-based and easily scalable intervention.

## Data availability statement

The raw data supporting the conclusions of this article will be made available by the authors, without undue reservation.

## Ethics statement

The studies involving human participants were reviewed and approved by Jewish General Hospital Research Ethics Committee. Registered: ClinicalTrials.gov, #NCT04523610. Written informed consent for participation was not required for this study in accordance with the national legislation and the institutional requirements.

## Author contributions

Conceptualization and methodology: SBu, SR, BV, NS, HS, and ED. Analysis and data curation: CR, PL, BV, HS, SR, C-LS, and KC. Draft preparation and writing and editing: HS, PL, BV, CR, and SR. Project administration and management: SBu, SR, BV, NS, HS, ED, PL, KC, CR, AG, FR, MI, MB, JG, JS, CW, RN, CH, IR, AS, and CL. Funding acquisition: SR, KL, HS, JG, KC, SBu, and JS. All authors have substantially contributed to the preparation, critical review, commentary revision, and approval of the manuscript.

## Funding

This work was funded by CIHR Grant# PJ8-169696, CIHR Grant # PJT-175191, FRQS - Grant# 2022-VIAP-308195, JGH Foundation, and Charitable donations from the Doggone Foundation.

## Conflict of interest

Author SR receives a salary award from the Fonds de Recherche de Québec-Santé (FRQS), is a steering committee member for AbbVie, and is a shareholder of Aifred Health. HS has a CIHR fellowship award, MITACS fellowship award, and AGE-WELL award. Author EM receives salary support from the Fond de recherche Santé Québec. Author SK has received research support from Brain and Behavior Foundation, National institute on Aging, BrightFocus Foundation, Brain Canada, Canadian Institute of Health Research, Canadian Consortium on Neurodegeneration in Aging, Centre for Ageing and Brain Health Innovation, Centre for Addiction and Mental Health, an Academic Scholars Award from the Department of Psychiatry, University of Toronto, and Equipment support from Soterix Medical. Author SBo receives funds from the Canada research Chairs program and various provincial and federal granting agencies. He is president of, and owns equity in, Cliniques et Development In Virtuo; a company that distributes virtual reality environments. Conflicts of interest are managed under UQO's conflicts of interest policy. The remaining authors declare that the research was conducted in the absence of any commercial or financial relationships that could be construed as a potential conflict of interest.

## Publisher's note

All claims expressed in this article are solely those of the authors and do not necessarily represent those of their affiliated organizations, or those of the publisher, the editors and the reviewers. Any product that may be evaluated in this article, or claim that may be made by its manufacturer, is not guaranteed or endorsed by the publisher.

## References

[1] Courtin E Knapp M. Social isolation, loneliness and health in old age: a scoping review. Health Soc Care Community. (2017) 25:799–812. 10.1111/hsc.12311 26712585

[2] Donovan NJ Blazer D. Social isolation and loneliness in older adults: review and commentary of a national academies report. Am J Geriatr Psychiatry. (2020) 28:1233–44. 10.1016/j.jagp.2020.08.005 32919873 PMC7437541

[3] Tomaka J Thompson S Palacios R. The relation of social isolation, loneliness, and social support to disease outcomes among the elderly. J Aging Health. (2006) 18:359–84. 10.1177/0898264305280993 16648391

[4] Cacioppo JT Hawkley LC Thisted RA. Perceived social isolation makes me sad: 5-year cross-lagged analyses of loneliness and depressive symptomatology in the Chicago health, aging, and social relations study. Psychol Aging. (2010) 25:453–63. 10.1037/a0017216 20545429 PMC2922929

[5] Luo Y Hawkley LC Waite LJ Cacioppo JT. Loneliness, health, and mortality in old age: a national longitudinal study. Soc Sci Med. (2012) 74:907–14. 10.1016/j.socscimed.2011.11.028 22326307 PMC3303190

[6] Golden J Conroy RM Bruce I Denihan A Greene E Kirby M. Loneliness, social support networks, mood and wellbeing in community-dwelling elderly. Int J Geriatr Psychiatry. (2009) 24:694–700. 10.1002/gps.2181 19274642

[7] Keesara S Jonas A Schulman K. Covid-19 and health care's digital revolution. N Engl J Med. (2020) 382:e82. 10.1056/NEJMp2005835 32240581

[8] Osenbach JE O'Brien KM Mishkind M Smolenski DJ. Synchronous telehealth technologies in psychotherapy for depression: a meta-analysis. Depress Anxiety. (2013) 30:1058–67. 10.1002/da.22165 23922191

[9] Rees CS Maclaine E. A systematic review of videoconference-delivered psychological treatment for anxiety disorders. Aust Psychol. (2015) 50:259–64. 10.1111/ap.1212234898445

[10] Preston C Moore S. Ringing the changes: the role of telephone communication in a helpline and befriending service targeting loneliness in older people. Ageing Soc. (2019) 39:1528–51. 10.1017/S0144686X18000120

[11] Cattan M Kime N Bagnall AM. The use of telephone befriending in low level support for socially isolated older people–an evaluation. Health Soc Care Community. (2011) 19:198–206. 10.1111/j.1365-2524.2010.00967.x 21114564

[12] Lurie N Carr BG. The role of telehealth in the medical response to disasters. JAMA Intern Med. (2018) 178:745–6. 10.1001/jamainternmed.2018.1314 29710200

[13] Hargittai E Dobransky K. Old Dogs, New clicks: digital inequality in skills and uses among older adults. Can J Commun. (2017) 42:195–212. 10.22230/cjc.2017v42n2a3176

[14] Neves BB Amaro F. Too old for technology? How the elderly of Lisbon use and perceive ICT. J Community Inf . (2012) 8. 10.15353/joci.v8i1.3061

[15] Dias A Azariah F Anderson SJ Sequeira M Cohen A Morse JQ. Effect of a lay counselor intervention on prevention of major depression in older adults living in low- and middle-income countries: a randomized clinical trial. JAMA Psychiatry. (2019) 76:13–20. 10.1001/jamapsychiatry.2018.3048 30422259 PMC6583466

[16] Dikaios E Sekhon H Allard A Vacaflor B Goodman A Dwyer E. Connecting during COVID-19: a protocol of a volunteer-based telehealth program for supporting older adults' health. Front Psychiatry. (2020) 11:598356. 10.3389/fpsyt.2020.598356 33343425 PMC7738321

[17] Doraiswamy S Jithesh A Mamtani R Abraham A Cheema S. Telehealth use in geriatrics care during the COVID-19 pandemic-a scoping review and evidence synthesis. Int J Environ Res Public Health. (2021) 18:1755. 10.3390/ijerph18041755 33670270 PMC7918552

[18] Ezzati A Jiang J Katz MJ Sliwinski MJ Zimmerman ME Lipton RB. Validation of the Perceived Stress Scale in a community sample of older adults. Int J Geriatr Psychiatry. (2014) 29:645–52. 10.1002/gps.4049 24302253 PMC4013212

[19] Kroenke K Spitzer RL Williams JB. The PHQ-9: validity of a brief depression severity measure. J Gen Intern Med. (2001) 16:606–13. 10.1046/j.1525-1497.2001.016009606.x 11556941 PMC1495268

[20] Spitzer RL Kroenke K Williams JBW Löwe B. A brief measure for assessing generalized anxiety disorder: the GAD-7. Arch Intern Med. (2006) 166:1092–7. 10.1001/archinte.166.10.1092 16717171

[21] Ahorsu DK Lin CY Imani V Saffari M Griffiths MD Pakpour AH. The fear of COVID-19 scale: development and initial validation. Int J Ment Health Addict. (2022) 20:1537–45. 10.1007/s11469-020-00270-8 32226353 PMC7100496

[22] Hanusz Z Tarasińska J. Normalization of the Kolmogorov–Smirnov and Shapiro–Wilk tests of normality. Biom Lett. (2015) 52:85–93. 10.1515/bile-2015-0008

[23] Mishra P Singh U Pandey CM Mishra P Pandey G. Application of student's t-test, analysis of variance, and covariance. Ann Card Anaesth. (2019) 22:407–11. 10.4103/aca.ACA_94_19 31621677 PMC6813708

[24] INSPQ. Ligne du temps COVID-19 au Québec. INSPQ. (2022). Available online at: https://www.inspq.qc.ca/covid-19/donnees/ligne-du-temps (accessed March 18, 2022).

[25] Su Z McDonnell D Wen J Kozak M Abbas J Šegalo S. Mental health consequences of COVID-19 media coverage: the need for effective crisis communication practices. Glob Health. (2021) 17:4. 10.1186/s12992-020-00654-4 33402169 PMC7784222

[26] López Peláez A Marcuello-Servós C Castillo de. Mesa J Almaguer Kalixto P. The more you know, the less you fear: reflexive social work practices in times of COVID-19. Int Soc Work. (2020) 63:746–52. 10.1177/0020872820959365

[27] Domènech-Abella J Mundó J Haro JM Rubio-Valera M. Anxiety, depression, loneliness and social network in the elderly: longitudinal associations from the Irish Longitudinal Study on Ageing (TILDA). J Affect Disord. (2019) 246:82–8. 10.1016/j.jad.2018.12.043 30578950

[28] Freedman A Nicolle J. Social isolation and loneliness: the new geriatric giants: approach for primary care. Can Fam Physician. (2020) 66:176–82. 32165464 PMC8302356

[29] Mistry R Rosansky J McGuire J McDermott C Jarvik L UPBEAT Collaborative Group. Social isolation predicts re-hospitalization in a group of older American veterans enrolled in the UPBEAT program unified psychogeriatric biopsychosocial evaluation and treatment. Int J Geriatr Psychiatry. (2001) 16:950–9. 10.1002/gps.447 11607938

[30] Steptoe A Shankar A Demakakos P Wardle J. Social isolation, loneliness, and all-cause mortality in older men and women. Proc Natl Acad Sci USA. (2013) 110:5797–801. 10.1073/pnas.1219686110 23530191 PMC3625264

[31] Katon WJ Lin E Russo J Unutzer J. Increased medical costs of a population-based sample of depressed elderly patients. Arch Gen Psychiatry. (2003) 60:897–903. 10.1001/archpsyc.60.9.897 12963671

[32] Lim KL Jacobs P Ohinmaa A Schopflocher D Dewa CS. A new population-based measure of the economic burden of mental illness in Canada. Chronic Dis Can. (2008) 28:92–8. 10.24095/hpcdp.28.3.02 18341763

[33] Zivin K Wharton T Rostant O. The economic, public health, and caregiver burden of late-life depression. Psychiatr Clin North Am. (2013) 36:631–49. 10.1016/j.psc.2013.08.008 24229661 PMC4024243

[34] Alami H Lehoux P Fleet R Fortin JP Liu J Attieh R. How can health systems better prepare for the next pandemic? Lessons learned from the management of COVID-19 in Quebec (Canada). Front Public Health. (2021) 9:671833. 10.3389/fpubh.2021.671833 34222176 PMC8249772

